# Thermally activated coupling between protein vibrations and interfacial water dynamics revealed by terahertz spectroscopy

**DOI:** 10.1016/j.isci.2026.115981

**Published:** 2026-05-25

**Authors:** Abhishek K. Singh, Nguyen Q. Vinh

**Affiliations:** 1 Department of Physics and Center for Soft Matter and Biological Physics, Virginia Tech, Blacksburg, VA 24061, USA

**Keywords:** Spectroscopy, Biophysics, Protein physics

## Abstract

Proteins exhibit complex dynamics that are strongly governed by their hydration environment. At the protein-water interface, strong electrostatic fields and topological confinement generate a heterogeneous landscape where water molecules exhibit distinct orientational behaviors. Broadband terahertz dielectric spectroscopy provides direct insight into the temperature-dependent coupling between protein collective vibrations and interfacial water dynamics. Using aqueous ubiquitin as a model system, three populations of loosely bound, tightly bound, and bulk water are identified. Tightly bound water exhibits an anomalous increase in dielectric strength with increasing temperature, in contrast to the expected trend observed for bulk and loosely bound water. This behavior correlates with enhanced protein vibrations, revealing a thermally activated vibrational, orientational coupling mechanism. Arrhenius analysis quantifies distinct activation energies and attempt-frequencies of each water population, demonstrating that protein vibrations actively modulate interfacial water behavior. These findings present a mechanistic framework for hydration-mediated biomolecular function and highlight terahertz spectroscopy as a unique tool to probe protein-water interactions.

## Highlights

• Broadband terahertz spectroscopy resolves the tightly bound water layer around protein
• Low-frequency protein vibrations are thermally activated
• Protein vibration-water orientational coupling observed at the interface
• Distinct attempt frequencies and energy barriers govern water relaxation modes

## Graphical abstract

**Figure:**
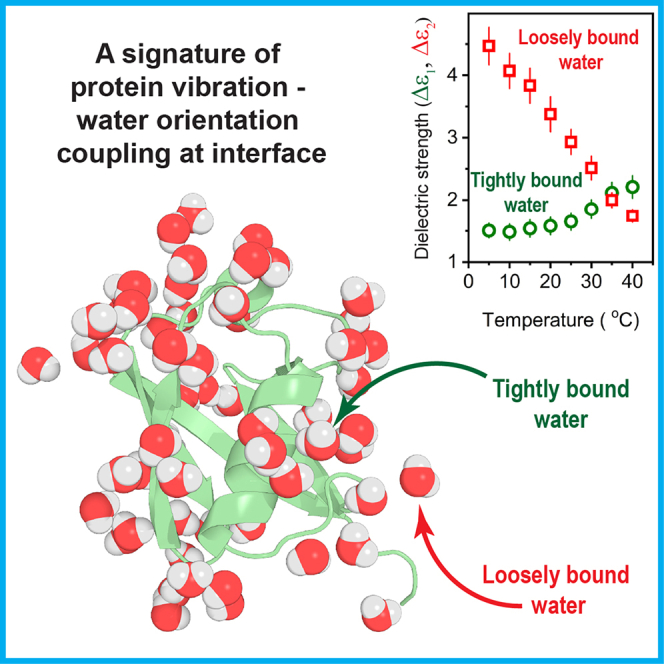

## Introduction

Proteins exist and function in aqueous environments, where water plays an active and complex role in their biological activities. Specifically, interactions between proteins and water are fundamental to protein structure, stability, dynamics, and catalytic functions, as water mediates hydrogen-bonding networks, electrostatic interactions, and participates in lubrication and plasticity for conformational motions.^1^^,^^2^^,^^3^ Around proteins, water molecules form structured hydration shells, where hydrogen bonding, electrostatic interactions, and local confinement restrict molecular mobility while simultaneously influencing protein flexibility.^4^^,^^5^^,^^6^ These interfacial water molecules act as dynamic regulators of biological activity, modulating energy landscapes, stabilizing native folds, and mediating essential processes such as signaling, enzymatic reactions, and molecular recognition events.^1^^,^^2^^,^^7^^,^^8^

Proteins in aqueous environments are inherently dynamic, undergoing atomic fluctuations across multiple timescales that are essential to their biological functions.^9^^,^^10^^,^^11^^,^^12^^,^^13^^,^^14^^,^^15^^,^^16^^,^^17^^,^^18^ Among these, low-frequency collective vibrational modes, including hinge motions, domain fluctuations, and correlated side-chain rearrangements, occur in the terahertz frequency range and are central to protein adaptability.^19^^,^^20^^,^^21^^,^^22^ These vibrational modes extend beyond the protein core into surrounding hydration layers, where they modulate the structure and dynamics of interfacial water.^23^^,^^24^^,^^25^^,^^26^^,^^27^^,^^28^^,^^29^^,^^30^^,^^31^^,^^32^ Hydration water, particularly the tightly bound component at the interface, exhibits unique orientational dynamics that differ substantially from bulk water and play a critical role in maintaining biological functions under varying physiological conditions.^12^^,^^13^^,^^24^^,^^25^^,^^32^^,^^33^^,^^34^^,^^35^

Despite growing recognition of the importance of hydration dynamics, it remains unclear how proteins dynamically influence their hydration shells beyond static structural effects.^7^^,^^23^^,^^25^^,^^27^ A central open question is whether collective protein motions can modulate the reorientation kinetics of interfacial water, establishing a feedback loop between protein flexibility and hydration. Terahertz absorption spectroscopy studies suggest that such coupling may occur, but its extent is not yet resolved in current models of biomolecular hydration.^7^ Moreover, studies have shown a correlation between protein backbone flexibility and hydration water diffusion, indicating a possible feedback loop between protein motions and surrounding water.^36^ However, this dynamic reciprocity is still under-characterized, particularly at physiological temperatures and in functionally relevant protein states. This gap is especially significant in the context of biological processes that depend on the fine-tuning of local dynamics, such as allosteric transitions, protein folding, temperature sensing, and hydration-dependent catalysis.^37^

To address this, we employ a high-precision broadband megahertz to terahertz spectroscopy to simultaneously capture the orientational relaxation of hydration water and collective vibrational responses of hydrated proteins across a wide temperature range. Aqueous ubiquitin solutions are used as a model system for resolving three distinct water populations, bulk, loosely bound, and tightly bound, and for tracking how their reorientation dynamics respond to temperature-induced changes in protein motion. An effective medium model is further applied to differentiate dielectric contributions from water molecules tightly associated with the protein surface. Building on previous studies on lysozyme, serum albumin and myoglobin, we apply Arrhenius analysis to determine the activation energies and attempt frequencies that govern protein hydration dynamics. This method allows us to quantify thermal adaptation processes and identify distinct dynamical regimes at the protein—water interface. Additionally, this study reveals a coupling between protein vibrational modes and the rotational relaxation of tightly bound water, which has not been previously addressed. In combination, the megahertz-to-gigahertz measurements resolve the orientational relaxation processes of hydration water, while terahertz spectroscopy captures low-frequency collective vibrational modes of hydrated proteins, directly linking hydration dynamics to protein functions.^25^^,^^27^^,^^34^^,^^38^

## Results and discussion

Dielectric spectra of an aqueous protein solution across megahertz to terahertz frequencies reveal the dynamic response associated with various *inter* and *intra*-molecular relaxation modes of proteins and surrounding water molecules. Specifically, rotational tumbling motions of proteins typically occur at frequencies around 100 kHz to 10 MHz,^14^^,^^31^^,^^39^ while orientational relaxation processes of water in the solution including tightly bound, loosely bound water, and bulk water take place at higher frequencies, extending into the gigahertz frequency region.^13^^,^^31^^,^^39^ In this context, we categorize water molecules into several groups. Tightly bound water molecules interact strongly and directly with the protein surface, whereas loosely bound water molecules, which experience weaker interactions or are not in direct contact with the surface, form an outer hydration layer. These loosely bound water molecules dynamically exchange with tightly bound water and exhibit relaxation behaviors approaching those of bulk water. Terahertz spectroscopy, in particular, captures the dielectric response associated with collective vibrational motions of hydrated proteins.^13^^,^^22^^,^^23^

**Figure 1:**
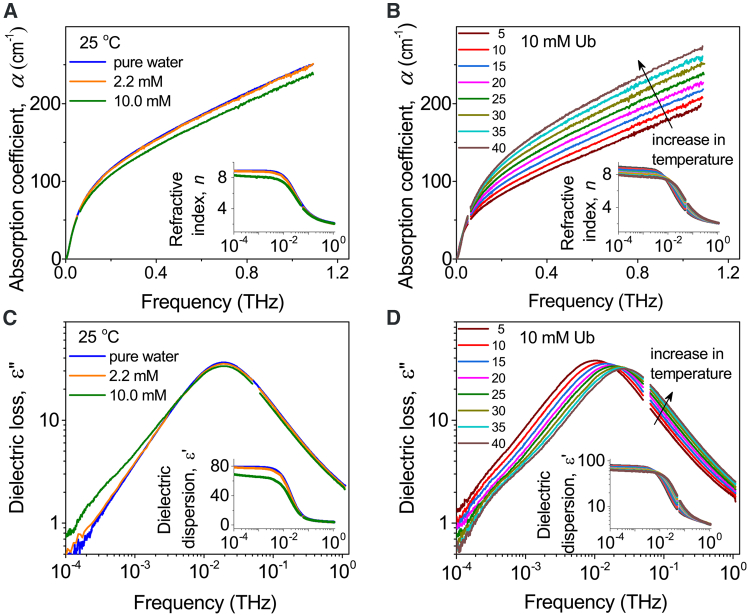
Broadband megahertz to terahertz response reveals hydrated protein dynamics (A) Absorption coefficient and refractive index spectra are collected for pure water, 2.2 and 10.0-mM ubiquitin solutions at 25°C. (B) Absorption coefficient and refractive index spectra for 10.0-mM ubiquitin solution at different temperatures from 5 to 40°C. (C and D) The corresponding dielectric dispersion, $\varepsilon^{\prime }\left(\nu \right)\text{,}$ and dielectric loss, $\varepsilon^{\prime \prime }\left(\nu \right)$, are determined from the absorption and refractive index measurements.

Using the frequency-domain megahertz-to-terahertz spectrometer, we simultaneously characterized the absorption coefficient and refractive index spectra, *α*(ν) and *n*(ν), respectively, for 2.2 and 10.0-mM ubiquitin solutions along with pure water (Figure 1A). The complex dielectric response of a protein solution is dominated by the main collective orientational mode of bulk water, which has a characteristic relaxation time of about 8.27 ps (i.e., 19.25 GHz) at 25°C. In contrast, a lower dielectric response corresponding to hydration water has been observed in the sub-gigahertz frequency region. Also shown in the figure are the absorption coefficient and refractive index spectra at temperatures from 5^o^C to 40^o^C (Figure 1B). Both measured properties show notable and systematic behaviors for concentration and temperature dependences.^13^^,^^24^^,^^31^^,^^34^ Since proteins have a significantly low absorbance compared to the highly absorbing water at the measured frequencies, the total absorbance of the solutions decreases with increasing protein concentration (Figure 1A). Using the absorption coefficient and refractive index spectra, both the real and imaginary parts, $\epsilon^{\prime }\left(\nu \right)$ and $\epsilon^{\prime \prime }\left(\nu \right)\text{,}$ respectively, of the complex dielectric function, $\epsilon^{*}\left(\nu \right)$, can be estimated at the measured temperatures (Figures 1C and 1D), as described in previous studies.^12^^,^^13^^,^^32^^,^^40^ The temperature variation influences the dynamic response of the aqueous protein solutions, causing the entire spectra to shift systematically to higher frequencies as the temperature increases.

### Dynamics and structure of hydration water around ubiquitin

**Figure 2:**
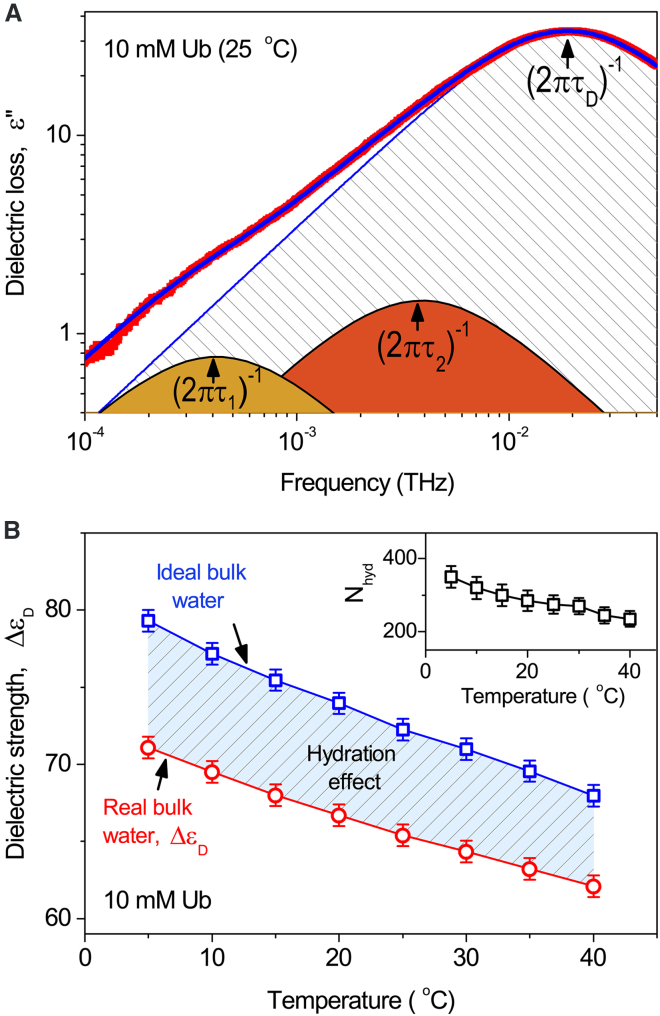
Characterization of hydration shells around ubiquitin using megahertz to gigahertz dielectric response (A) Dielectric response of a 10.0-mM ubiquitin solution at 25°C was deconvoluted into three components using a three-Debye model, revealing orientational dynamics of bulk water, $\tau_{D}$, tightly bound water, $\tau_{1}$, and loosely bound water, $\tau_{2}$. (B) Dielectric strength, $\Delta \epsilon_{D}$, of “*real bulk water*” obtained from Equation 1 is shown against temperature (in red). Also shown is the theoretical estimation of “*ideal bulk water*,” considering that all water molecules in the solution follow the pure water orientational dynamics, $\tau_{D}$ (in blue). The estimated hydration number as a function of temperature is plotted in the inset. Error bars represent standard uncertainties from nonlinear least-squares fitting of the three-Debye model for dielectric strengths.

The orientational motion of water molecules around protein surface is kinetically retarded as compared to that in bulk water, which is characterized by a relaxation time of 8.27 ps at 25°C. This slowdown is primarily driven by the electrostatic potential of the protein surface, which strongly influences the structure and dynamics of interfacial water. However, this effect decays rapidly with increasing distance from the surface. As a result, water molecules near the protein surface experience varying electrostatic potentials, leading to diverse dynamical behaviors. Additionally, the electrostatic potential of the protein surface is not uniform, giving rise to a heterogeneous environment of water molecules in this region. To capture this complexity, we apply a Debye relaxation model^25^^,^^31^^,^^39^ with three distinct components, including tightly bound, loosely bound, and bulk water, expressed as,

(Equation 1) $$\epsilon_{\text{sol}}^{*}\left(\nu \right)-\epsilon_{\infty }=\frac{\epsilon_{S}-\epsilon_{1}}{1+i2\pi \nu \tau_{1}}+\frac{\epsilon_{1}-\epsilon_{2}}{1+i2\pi \nu \tau_{2}}+\frac{\epsilon_{2}-\epsilon_{\infty }}{1+i2\pi {\nu \tau }_{D}}+\frac{\sigma }{2\pi \nu \epsilon_{0}}\text{,}$$

where *τ*_1_, *τ*_2_, and *τ*_D_ are the relaxation times, and the corresponding numerators, $\epsilon_{S}-\epsilon_{1}=\Delta \epsilon_{1}$, $\epsilon_{1}-\epsilon_{2}=\Delta \epsilon_{2}$, and $\epsilon_{2}-\epsilon_{\infty }=\Delta \epsilon_{D}$ are the dielectric strengths of the tightly bound, loosely bound, and bulk water, respectively, $\epsilon_{0}$ is the permittivity of the vacuum, and σ is the electrical conductivity of the solution. The three-Debye representation provides a macroscopic description of the heterogeneous dynamics of the protein-water system. Each Debye term represents an effective dynamical sub-ensemble at thermal equilibrium, characterized by relaxation time *τ*ᵢ and dielectric strength $\Delta \epsilon_{i}$. The temperature-dependent parameters, *τ*_i_(T) and $\Delta \epsilon_{i}$ (T), therefore reflect how spectral weight is distributed among bulk, loosely bound, and tightly bound environments as the equilibrium distribution of water dynamics changes with temperature. Similar multi-Debye decompositions are widely used to describe the heterogeneous relaxation behavior of aqueous and biomolecular solutions.^25^^,^^31^^,^^41^^,^^42^^,^^43^ The global reorientation dynamics of hydrated protein, which effectively includes both the “*bare*” protein and its associated hydration water, typically occur on timescales corresponding to frequencies between 10 kHz and 10 MHz, well below the frequency range of our measurements. Therefore, this slow rotational process was excluded from our analysis. An example of the fit to Equation 1 for the dielectric loss, $\epsilon^{\prime \prime }\left(\nu \right)\text{,}$ is shown in Figure 2A for a 10.0 mM ubiquitin solution at 25°C.

The relaxation times obtained from the fitting are 331 ± 30, 42 ± 4, and 8.2 ± 0.2 ps, for tightly bound, loosely bound, and bulk water, respectively. As expected, the characteristic relaxation time for bulk water in the solution remains close to 8.27 ps, consistent with the value reported for pure water.^44^^,^^45^ However, the dynamics of water molecules in hydration shells are kinetically retarded compared to those of bulk water, with retardation factors of approximately 40 and 5 for tightly bound and loosely bound water, respectively. The observations conform well to previous studies on different protein solutions,^13^^,^^31^^,^^46^^,^^47^ which employed various different methods to characterize water dynamics across a wide range of timescales, providing a comprehensive perspective on bound water dynamics. For instance, nuclear magnetic resonance (NMR) studies showed retardation factors of 2–5 for bound water around protein.^48^ In a separate NMR study, Wüthrich et al.,^49^ reported that relaxation times of hydration water must be less than 300 ps. Earlier dielectric spectroscopy investigations by Mashimo,^50^ Pethig,^51^ Grant,^52^ and co-workers observed relaxation times of water molecules ranging from 10 ps to 10 ns, including a relaxation time of ∼40 ps. Additionally, time-dependent fluorescence Stokes shift observations also reported slow relaxation components with retardation factors between 10 and 100.^4^^,^^53^^,^^54^^,^^55^ The close correspondence across independent techniques highlights the robustness of current three-Debye analysis in resolving the multiscale dynamics of hydration water.

As protein concentration increases, the dielectric response increases in the sub-gigahertz region, while the main peak of the dielectric loss at 19.25 GHz decreases (Figure 1C). This behavior originates from the presence of protein and the interaction between protein surface and water molecules forming hydration layers. Proteins exhibit low absorption at these frequencies and replace strongly absorbing water molecules in the solution, resulting in a net decrease in the total absorbance and dielectric loss. Additionally, a substantial fraction of water molecules near the protein surface is kinetically slowed down due to electrostatic potential of the protein surface. These slowed water molecules are no longer part of bulk water but instead contribute to hydration water, resulting in an increase in the dielectric response in the sub-gigahertz frequency region.

Temperature influences the dynamics of water and protein in the solution, including the dielectric strength and relaxation time of these molecules. Specifically, the dielectric strength at 19.25 GHz of bulk water in the solution reduces gradually as the temperature increases (Figure 2B). Theoretically, the partial specific volume of water in the protein solution can be used to calculate the dielectric strength of “*ideal bulk water*,” assuming that all water molecules behave as bulk water, characterized by the relaxation time $\tau_{D}$. However, the experimentally determined dielectric strength of bulk water, $\Delta \epsilon_{D}$, extracted from Equation 1 at each temperature, is lower than the theoretical value and corresponds to the dielectric response of the “*real bulk water*” in the protein solution (Figure 2B). This discrepancy is attributed to the “*hydration effect*,” indicating that not all water molecules at a given temperature contribute to the bulk water in the solution. These water molecules are kinetically retarded and participate in the hydration water surrounding the protein. The number of such molecules can be determined based on the reduction in the dielectric strength of bulk water, and identified as the “*hydration number*,” in the form,^24^^,^^40^

(Equation 2) $$N_{\text{hyd}}\left(T\right)=\frac{c_{w}-\frac{\Delta \epsilon_{w}\left(T\right)}{\Delta \epsilon_{\text{pure}}\left(T\right)}{\;c}_{\text{pure}}}{c}$$

where *c*_pure_ is the molarity of pure water, $c_{w}$ is the water concentration in the solution, $\Delta \epsilon_{\text{pure}}$ (*T*) is the dielectric strength corresponding to the ideal bulk water having the relaxation time $\tau_{D}$ at temperature *T*, $\Delta \epsilon_{w}\left(T\right)$ is the dielectric strength of bulk water in the protein solution, and *c* is the concentration of protein in the solution. The total hydration sheath consists of approximately 275 water molecules per ubiquitin protein at 25°C, including tightly bound and loosely bound water molecules. The hydration numbers at different temperatures are shown in Figure 2B, inset. A decrease in *N*_hyd_ is observed at elevated temperatures, indicating the hydration structure around protein is weakened by temperature. In this context, $N_{\text{hyd}}\left(T\right)$ represents an equilibrium measure of the average number of water molecules participating in hydration shell at temperature T, rather than a static count of permanently bound water molecules. The observed decrease in *N*_hyd_ at higher temperature (Figure 2B, inset) therefore reflects a redistribution of the dipolar response from slower hydration-water modes toward faster, bulk-like orientational dynamics.

**Figure 3:**
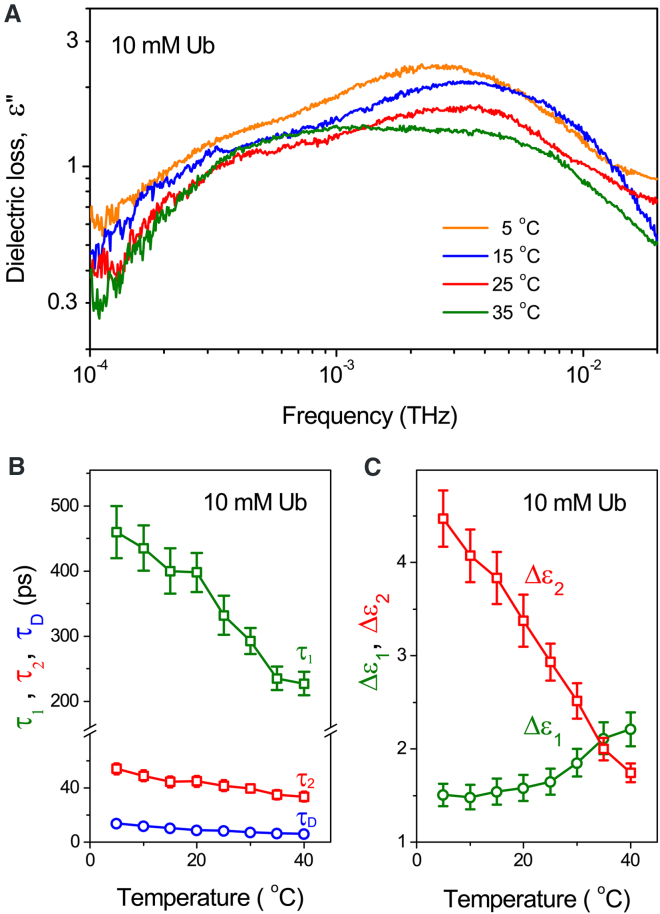
Hydration water dynamics are strongly coupled to temperature fluctuations (A) The dielectric contribution of bound water molecules, including the tightly bound and loosely bound water, is estimated by subtracting the bulk water contribution from the total dielectric response of a 10.0-mM ubiquitin solution. (B and C) Relaxation times and dielectric response, respectively, of tightly bound ($\tau_{1}$, $\Delta \epsilon_{1}$), loosely bound ($\tau_{2}$, $\Delta \epsilon_{2}$), and bulk water ($\tau_{D}$) are shown against temperature. Error bars in (B) and (C) represent standard uncertainties obtained directly from nonlinear least-squares fitting of the three-Debye model parameters for dielectric strength and relaxation time.

To gain a comprehensive understanding of the behavior of water molecules around protein surface at different temperatures, the contribution from hydration water to the total dielectric response has been analyzed separately (Figure 3). The dielectric contribution from all bound water molecules was obtained by subtracting the dielectric response of bulk water from the total dielectric response at the probed temperature (Figure 3A). As shown, the dynamics of hydration water consist of two components with relaxation times of $\tau_{1}$ and $\tau_{2}$, corresponding to tightly bound and loosely bound water, respectively (Figure 2A). The relaxation times and corresponding dielectric strengths are plotted as a function of temperature in Figure 3. A gradual decrease in the relaxation times with increasing temperature has been observed for both relaxation modes. Concurrently, a decrease in the dielectric strength of loosely bound water, $\Delta \epsilon_{2}$, was also identified, following a similar trend to bulk water. This behavior indicates a reduced orientational correlation among water molecules due to elevated thermal fluctuations at higher temperatures. Interestingly, the dielectric response of tightly bound water, $\Delta \epsilon_{1}$, increases as the temperature increases. This observation suggests rotational-vibrational coupling at the protein-water interface, which is discussed later.

### Terahertz collective vibrational motions of hydrated proteins

Terahertz spectroscopy provides valuable insights into conformational changes of hydrated proteins, which are correlated to their functional mechanisms in an aqueous environment. Conducting such experiments has been challenging recently due to the strong absorption of water in the gigahertz to terahertz frequency region. By employing our spectrometer with a high dynamic range, we have precisely measured the refractive index and absorption coefficient of aqueous protein solutions in this frequency region. This capability enables the investigation of collective vibrational modes of hydrated proteins as well as simultaneously determines water molecules strongly bound to the protein surface.

**Figure 4:**
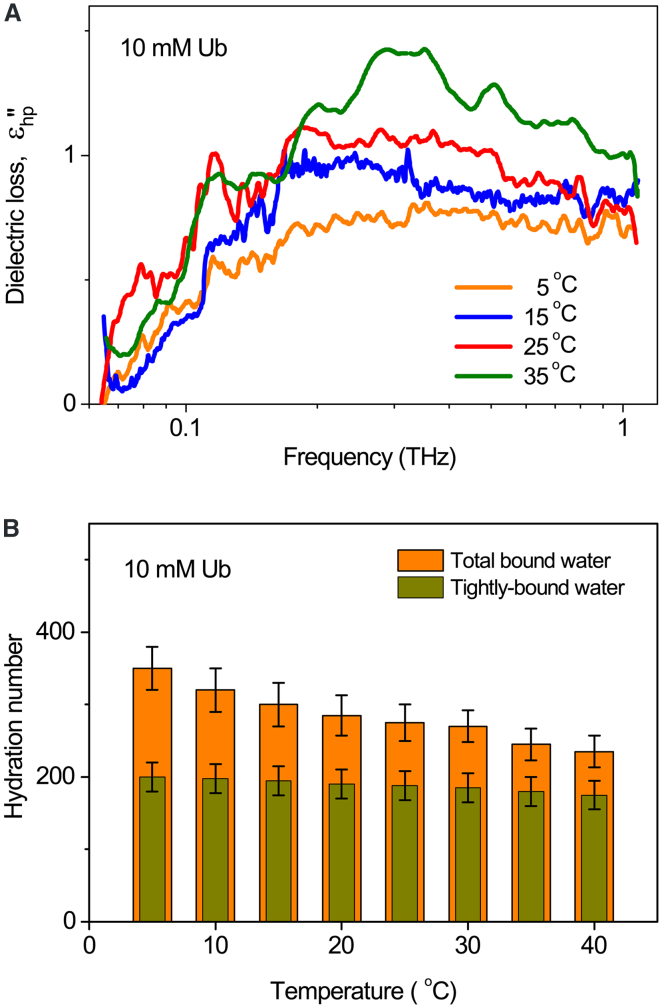
Hydrated proteins exhibit thermally activated terahertz vibrational modes (A) The dielectric loss corresponding to hydrated protein, $\varepsilon_{\text{hp}}^{\prime \prime }\left(\nu \right)$, originating from terahertz collective vibration modes at different temperatures show that the modes are thermally activated and become more pronounced at elevated temperatures. (B) The number of tightly bound water molecules (in dark yellow) and the total number of water molecules bound to a protein (in orange) vary with temperature.

To elucidate the collective vibrational motions and the number of tightly bound water molecules embedded in hydrated proteins, an effective-medium approximation has been employed. In this approach, protein solutions are modeled as a composite mixture of water and hydrated proteins, each characterized by their respective complex dielectric responses, $\epsilon_{w}^{*}$ and $\epsilon_{\text{hp}}^{*}$. Since terahertz wavelengths are several orders of magnitude larger than the protein size, the protein solution can be considered as an effective-homogeneous medium. Several effective-medium models have been developed for a variety of composite systems.^56^^,^^57^^,^^58^^,^^59^ In particular, the Bruggeman effective-medium model is appropriate for systems with a high dielectric contrast between protein and water and applicable over a broad concentration range.^56^^,^^57^^,^^59^ The effective-medium approach does not resolve protein shape anisotropy or local variations in hydration structure; thus, the extracted hydrated-protein permittivity and hydration numbers represent spatially averaged, effective quantities rather than atomistic structural features. The approximation is employed under following assumptions: (i) the globular protein is considered as a spherical macromolecule with an effective radius *R*_p_. (ii) The protein surface strongly interacts with tightly bound water, which is considered as a part of the hydrated protein and participates in its collective vibrational modes at terahertz frequencies. Therefore, hydrated proteins with an effective radius of (*R*_p_ + *d*) are considered to be solvated into the solution, where *d* is thickness of the tightly bound water layer. And, (iii) finally, water molecules outside hydrated proteins behave as bulk water. Under these assumptions, the Bruggeman approximation was used in the form,

(Equation 3) $$f_{\text{hp}}\frac{\epsilon_{\text{hp}}^{*}-\epsilon_{\text{sol}}^{*}}{\epsilon_{\text{hp}}^{*}+{2\epsilon }_{\text{sol}}^{*}}+\left(1-f_{\text{hp}}\right)\frac{\epsilon_{w}^{*}-\epsilon_{\text{sol}}^{*}}{\epsilon_{w}^{*}+2\epsilon_{\text{sol}}^{*}}=0$$

where $f_{\text{hp}}=\left(N_{p}/V\right)\left(4\pi /3\right){\left(R_{p}+d\right)}^{3}$ is volume fraction of the hydrated protein, (*N*_p_/*V*) is the concentration of protein in the solution. The dielectric response of hydrated proteins is shown at different temperatures in Figure 4A. A simultaneous estimation of the number of tightly bound water molecules and the total bound water is provided for comparison in Figure 4B. Analysis using the effective-medium approximation at terahertz frequencies reveals that more than half of the total hydration water molecules directly interact with the protein surface, forming the tightly bound water layer around protein (Figure 4B). The terahertz collective vibrations of hydrated protein show a strong temperature dependence, signifying the fact that these collective modes are thermally activated. The broad terahertz dielectric loss peak position of the collective vibration spectrum shifts slightly to a higher frequency as the temperature increases. Correspondingly, the thermal fluctuations in the hydrated protein enhance with increase in temperature, giving rise to a net increase in the amplitude of the dielectric response due to the terahertz collective vibrational modes.

### Temperature dependence of water dynamics at protein-water interface

Structural changes in protein, particularly in the context of hydration water, are affected by the interactions of water molecules with the protein surface as well as the temperature, governing the folding and stability of protein. Hydration water forms a dynamic shell around the protein, stabilizing its structure through hydrogen bonding and mediating hydrophobic interactions. As temperature changes, these water-protein interactions are altered, thereby impacting the overall structural integrity of the protein. Temperature-induced structural changes in hydrated proteins can be observed through collective vibrational modes in the terahertz frequency region. Specifically, at low temperatures the large-scale terahertz vibrational modes are restricted, helping to stabilize protein conformations. As temperature increases, these terahertz collective vibrational motions become more pronounced, reflecting weakened water-protein interactions and protein destabilization, often resulting in unfolding at elevated temperatures. Figure 4A shows that the terahertz collective vibrational motions of hydrated proteins become more pronounced at elevated temperatures. On the other hand, no significant changes in the tightly bound water molecules are observed (Figure 4B). This behavior suggests that no significant structural changes, such as denaturation, occur in the studied temperature range.

The total number of water molecules in the hydration sheath is highly temperature-dependent, reducing at high temperatures as loosely bound water is released from the hydration shell. These loosely bound water molecules interact weakly with the protein surface, and the interaction is disrupted at elevated temperatures due to increased thermal fluctuations. Consequently, the reduction in the total number of hydration water molecules (Figure 2B) is primarily due to the decrease in loosely bound water molecules with increasing temperature. Within the three-Debye framework, this release corresponds to a reduction in the dielectric strength of the loosely bound component and a compensating increase in the effective bulk-like contribution, i.e., an equilibrium shift of spectral weight between these dynamical regimes.

Thermal fluctuations of water molecular dipoles increase with temperature, causing a reduction in their net orientation. This is evidenced by the gradual decrease in the dielectric response of bulk water with increasing temperature (Figure 2B).^9^^,^^13^^,^^24^^,^^25^^,^^31^ A similar trend in dielectric strength is observed for loosely bound water (Figure 3A). Loosely bound water molecules, located in between bulk water and tightly bound water, exhibit reduced orientational correlation due to the presence of proteins, behaving as partially uncorrelated, bulk-like water. The thermal fluctuation of these water molecules enhances when the temperature increases, which is reflected in the reduction of the dielectric strength at elevated temperature. In contrast, the dielectric response of tightly bound water rises with temperature. Being highly enslaved to protein surface, the orientational relaxation of tightly bound water is governed by collective vibrational motions of the protein. At elevated temperatures, a pronounced increase in terahertz vibrational modes of hydrated proteins is observed (Figure 4A). This trend conforms well with the adaptation of protein to temperature variation, as seen in mean square deviations.^60^ We propose that the thermally activated collective motions of the protein weaken the protein-water interaction, leading to an increase in the net orientational polarization of water molecules. This behavior appears to be characteristic of the intermolecular coupling between protein vibrations and water rotations at the protein-water interface. The underlying fluctuations in the protein surface facilitate the orientational motion of water molecules, resulting in an increase in the dielectric strength of tightly bound water as temperature rises.

The relaxation rates of both pure and hydration water exhibit deviations from a simple Arrhenius behavior at low temperatures, where molecular reorganization becomes slower and more constrained, leading to complex relaxation processes.^24^ In this study, which focuses on temperatures above 0°C, the dynamics are largely thermally activated. In this temperature regime, the Arrhenius equation not only provides a reliable description of the relaxation processes but also offers valuable information of key parameters like attempt frequency and activation energy. These parameters are essential for understanding different types of water dynamics and the degree of molecular freedom in various biological and chemical environments.

**Figure 5:**
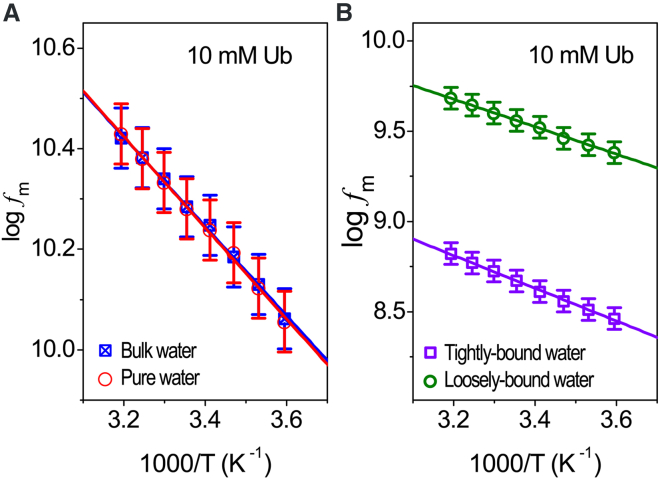
Orientational relaxation modes of water molecules in a 10.0-mM aqueous ubiquitin solution and pure water strongly depend on temperature, as shown in Arrhenius representation (A) The characteristic relaxation frequencies corresponding to bulk water relaxation, $\tau_{D}$, in 10.0-mM ubiquitin solution and pure water are shown against inverse temperature (1,000/T). (B) The characteristic relaxation frequencies corresponding to tightly bound, $\tau_{1}$, and loosely bound water, $\tau_{2}$, are plotted against inverse temperature, (1,000/T), for the 10.0-mM ubiquitin solution.

The temperature dependence of the relaxation time, τ, or equivalently, the frequency at the peak of dielectric loss, *f*_m_, associated with the orientational dynamics of water molecules in protein solutions, follows the Arrhenius relationship,^9^^,^^61^^,^^62^^,^^63^

(Equation 4) $$f_{m}=f_{0}\;\exp\left(-E_{\text{act}}/RT\right)$$

where *f*_m_ = 1/(2πτ), *E*_act_ is the activation energy barrier, and *f*_0_ is the pre-exponential factor representing the “*attempt frequency*.” The latter represents the number of reorientation attempts per second that a water molecule makes to overcome intermolecular restrictions imposed by hydrogen bonding. The extracted activation energies, *E*_act_, and pre-exponential factors, *f*_0_, corresponding to different orientation modes are summarized for a 10.0-mM ubiquitin concentration in Table 1. The temperature dependence of the peak position of the dielectric loss, *f*_m_ (=1/2πτ_m_), for the same solution is plotted against inverse temperature in Figure 5. Bulk water molecules in the solution, located far away from the protein surface, exhibit Arrhenius parameters similar to those of pure water (activation energy ∼17.7 kJ/mol and pre-exponential factor, log *f*_0_, of ∼13.5), indicating that the native tetrahedral hydrogen-bond network is maintained for bulk water in protein solutions.

**Table 1:** Protein-water interactions govern relaxation processes of hydration water

Orientational relaxation modes	log *f*_0_	*E*_act_ (kJ/mol)
Pure water	13.5	17.7
Bulk water (in protein solution)	13.4	17.6
Loosely bound water (in protein solution)	12.1	14.6
Tightly bound water (in protein solution)	11.7	17.3

Orientational relaxation modes of water in a 10.0-mM aqueous ubiquitin solution are strongly influenced by the protein-water interaction, as indicated by the attempt frequency, *f*_0_, and activation energy, *E*_act_.

Bound water molecules are significantly affected by the protein surface. Loosely bound water accumulated in outer hydration shells has weak interactions with the protein surface and disrupts the hydrogen-bond network of bulk water. With an activation energy of approximately 14.6 kJ/mol (Table 1), these water molecules exhibit lower correlated hydrogen bonding interactions compared to bulk water and tightly bound water. Loosely bound water, which is dynamically decoupled from the innermost hydration layer, experiences reduced orientational constraints. Consequently, these water molecules have faster reorientation times and weaker protein-water interactions.^7^^,^^13^^,^^25^^,^^64^^,^^65^^,^^66^^,^^67^ Additionally, the dielectric response and relaxation time of these water molecules decrease as the temperature increases, indicating the structural breaking effect of loosely bound water molecules. This behavior aligns with molecular simulations proposed by Persson et al.,^68^ for the orientational modes.

In contrast, tightly bound water molecules, forming the innermost hydration layer around the protein, exhibit stronger interactions with the protein surface and require a higher activation energy of 17.3 kJ/mol. This agrees with reports suggesting that tightly bound water molecules participate in direct hydrogen-bonding with protein residues, making them more rigid and requiring higher energy to reorient.^69^^,^^70^ Although the activation energy for tightly bound water molecules is similar to that of bulk water, the interaction mechanism differs. The activation barrier for bulk water is primarily driven by interactions between water molecules, whereas the barrier for tightly bound water arises from interactions with the protein surface, which influences both local water structure and dynamics.

The pre-exponential factor, *f*_0_, in the Arrhenius equation, often interpreted as the *attempt frequency*, represents the number of reorientation attempts water molecules make per second, exploring the behavior of different types of water molecules in different environments. For pure water (log *f*_0_ = 13.5), the molecules exhibit a high attempt frequency, trying to reorient approximately 10^13.5^ times per second. This suggests that in pure water, water molecules are constantly attempting to reorient within their dynamic hydrogen bond network. Despite the high attempt frequency to reorient, these molecules still need to overcome an activation energy of 17.7 kJ/mol to successfully change their orientation. Similarly, bulk water in an aqueous protein solution (log *f*_0_ = 13.4) also shows a high attempt frequency, making 10^13.4^ reorientation attempts per second. The activation energy for bulk water is similar at 17.6 kJ/mol, indicating that these molecules experience almost the same barrier to reorientation as pure water, as they are barely affected by the nearby proteins.

The pre-exponential factor of bound water molecules is influenced by the protein surface. Loosely bound water molecules located further from the protein surface have a lower attempt frequency (log *f*_0_ = 12.1), indicating that they try to reorient less frequently, approximately 10^12.1^ times per second. These molecules experience less constrained due to relatively weaker hydrogen bonding, both with the protein and with other water molecules, which is reflected in their lower activation energy of 14.6 kJ/mol. Although they have a lower attempt frequency, the lower energy barrier means that when they do, they require less energy to successfully reorient. This lower activation energy arises from the weaker and more transient hydrogen bonds they form.

Tightly bound water molecules, on the other hand, exhibit the lowest attempt frequency (log *f*_0_ = 11.7), trying to reorient only approximately 10^11.7^ times per second. These molecules are strongly bound to the protein surface through direct hydrogen bonds with charged protein residues, which restricts their motion and reduces the frequency of their reorientation attempts. Despite their lower attempt frequency, tightly bound water still has a relatively high activation energy of 17.3 kJ/mol. This reflects the strong interactions with the protein surface that must be overcome for these molecules to successfully reorient, making their dynamics slower and more constrained compared to loosely bound or bulk water.

While several studies have investigated hydration in protein solutions, many do not resolve the hydration dynamics into distinct components or report Arrhenius parameters for these processes. The ability to quantitatively separate these contributions highlights the advantage of broadband dielectric spectroscopy in uncovering the complexity of interfacial water dynamics across different protein systems. For example, a dielectric study on lysozyme solutions^39^ reported a single bound water relaxation (δ-relaxation) with an activation energy of 16.4–18.3 kJ/mol. In contrast, our analysis of ubiquitin resolves two kinetically distinct hydration layers, loosely bound and tightly bound water, each with distinct relaxation times, activation energies, and attempt frequencies. Notably, the tightly bound component exhibits an activation energy similar to the δ-process in lysozyme, but with a substantially lower attempt frequency, indicating more restricted orientational dynamics. These differences likely reflect both the intrinsic structural and surface differences between lysozyme and ubiquitin, and the fact that previous studies were unable to decompose the δ-relaxation into individual contributions, resulting in a composite signature. Crucially, resolving the tightly bound hydration layer has revealed salient features of interfacial water dynamics, including its unique energetic and kinetic properties and its coupling to protein vibrational modes. These findings contribute to a more detailed understanding of protein-water interactions and form a basis for future exploration of their functional relevance.

### Vibrational-mechanical coupling at the ubiquitin-water interface

Temperature-dependent dielectric analysis reveals that increasing temperature reduces the contribution of the loosely bound hydration water, while the tightly bound layer maintains a nearly constant population but exhibits increasing dielectric strength. This behavior indicates that interfacial water develops a stronger orientational response at higher temperatures, consistent with a softening of orientational constraints of water molecules at the protein surface.

In a standard electrostatic framework, protein-water interactions arise from dipole-dipole coupling, where permanent and induced charges on the protein surface create an inhomogeneous electrical field that partially orients nearby water molecules. The resulting slow dielectric response reflects the field-induced dipole alignment, protein-water dipolar cross-correlations, and exchange between bound and bulk water.^41^^,^^71^^,^^72^ With increasing temperature, rotational diffusion and exchange rates rise, reducing the degree of static dipolar alignment thereby decreasing the amplitude of the slow, strongly constrained component. While such models successfully capture the coexistence of bound and bulk water in protein solutions, they do not naturally explain the temperature-driven increase in the dielectric strength of tightly bound water when its population remains essentially constant, as observed for ubiquitin.

A more consistent explanation of the sub-terahertz dielectric response is vibrational-mechanical coupling between low-frequency protein motions and their hydration water. Theory and simulations have shown that proteins possess a dense spectrum of delocalized, low-frequency vibrational modes whose energies and lifetimes are strongly influenced by the solvent. These modes provide efficient channels for energy exchange between the protein and surrounding water.^73^^,^^74^ Terahertz and far-infrared experiments, supported by simulations, further demonstrate that these collective modes are strongly damped at the interface and give rise to correlated protein-water interactions extending over several hydration layers.^73^^,^^74^ Within this framework, thermally populated low-frequency modes in ubiquitin continuously modulate the interfacial hydrogen-bond structure, lowering rotational barriers for tightly bound water molecules without altering their overall population. Consequently, the increase in dielectric strength with temperature of tightly bound water reflects a softening of the interfacial orientational potential. Additionally, ultrafast vibrational experiments have strengthened this interpretation. Femtosecond pump-probe and infrared studies show rapid energy transfer from backbone vibrations to adjacent water, with amide-I modes relaxing significantly faster in H_2_O than in D_2_O.^75^^,^^76^ Follow-up work further shows that strong coupling to hydration water alters amide-I spectral features, demonstrating the hydration shell as an effective sink for vibrational energy.^77^

Accordingly, the tightly bound water contribution observed in sub-terahertz dielectric spectroscopy can be viewed as a macroscopic signature of coupled protein-water vibrational modes. The extracted activation energy reflects the reorganization cost of the interfacial hydrogen-bond network rather than a simple single-molecule rotational barrier. The increase in dielectric strength with temperature for tightly bound water molecules indicates that these coupled modes become increasingly effective at driving interfacial reorientation as protein vibrations are thermally populated. In this view, electrostatic dipole-dipole interactions establish the baseline interfacial polarization, while vibrational-mechanical modulation of the hydrogen-bond network, as supported by theories of energy flow in proteins and by pump-probe experiments,^73^^,^^74^^,^^75^^,^^76^^,^^77^ emerges as the dominant mechanism through which ubiquitin motions tune hydration-water orientation in the sub-terahertz regime.

Although our temperature-dependent analysis is performed on ubiquitin, the underlying mechanism is likely general. Previous megahertz-to-terahertz dielectric studies on myoglobin resolved bulk, loosely bound, and tightly bound hydration water with the total hydration number decreasing at elevated temperature due to the release of loosely bound water.^24^ Similar results from terahertz spectroscopy and other methods on myoglobin, lysozyme, and serum albumin show that proteins generally carry a dynamic hydration shell whose polarizability and spatial extent depend on surface electrostatics, hydrophobicity, and conformational state.^7^^,^^9^^,^^13^^,^^15^^,^^31^ Within this broader context, the increase of tightly bound dielectric strength with temperature observed for ubiquitin appears to be a protein-specific expression of a more general scenario where low-frequency protein motions modulate the interfacial hydrogen-bond structure, thereby tuning orientational response of hydration water. Systematic experiments and simulations across proteins with diverse surface charge patterns and flexibilities will be valuable for determining how broadly this vibrationally mediated protein-water coupling operates across protein systems.

In conclusion, this study establishes a mechanistic connection between protein collective motions and interfacial hydration dynamics. By integrating broadband terahertz dielectric spectroscopy with effective medium modeling, we quantitatively resolved bulk, loosely bound and tightly bound water populations surrounding ubiquitin, each exhibiting characteristic relaxation behaviors. Among these, tightly bound water exhibited an unexpected increase in dielectric strength with temperature, correlating with enhanced low-frequency protein vibrations. These results provide direct evidence for a bidirectional coupling between protein flexibility and hydration water, indicating that collective vibrational modes in protein extend into the interfacial hydration layer and modulate water reorientation kinetics in a thermally sensitive manner. Such coupling is essential for maintaining protein function and structural integrity. These findings enhance the understanding of protein hydration, demonstrating how structural dynamics of water are closely linked to protein stability and biological activity. The study highlights the potential of terahertz spectroscopy as a powerful tool for investigating protein-water interactions, with implications for areas such as protein folding, drug design, and the study of disease-related protein misfolding and aggregation.

### Limitations of the study

The present study establishes an experimental framework for probing protein-hydration coupling at terahertz frequencies, though several limitations should be noted. First, all measurements were performed on a model protein, ubiquitin. While the multi-Debye decomposition and effective-medium approach employed here can in principle be extended to other systems, it remains an open question whether the thermally activated orientational-vibrational coupling mechanism identified in this work is broadly applicable. Proteins with remarkably different surface characteristics, such as predominantly hydrophobic interfaces, strongly hydrophilic surfaces, or intrinsically disordered architectures, may exhibit distinct hydration dynamics and coupling behaviors. Resolving this question will require systematic comparisons across a set of proteins.

Second, the vibrational-mechanical coupling is inferred from macroscopic dielectric observables. While the results are consistent with thermally activated coupling between collective protein vibrational modes and the orientational dynamics of interfacial water, they do not directly resolve the underlying molecular processes. In particular, the present approach cannot disentangle the relative roles of electrostatic dipole interactions and changes in the interfacial hydrogen-bond network. Future studies combining atomistic molecular dynamics simulations with complementary ultrafast and surface-sensitive spectroscopic methods will be important for elucidating the microscopic origin of the observed coupling.

## Resource availability

### Lead contact

Further information and requests for resources should be directed to and will be fulfilled by the lead contact, Nguyen Q. Vinh (vinh@vt.edu).

### Materials availability

The material generated in this study was ubiquitin from bovine erythrocytes purchased from Sigma-Aldrich. All solutions were prepared in our laboratory following the procedures described in the Biological Samples and Sample preparation sections. No new or unique reagents or materials were generated in this work.

### Data and code availability

• This article does not report any original code. Custom Origin and MATLAB routines used for dielectric data analysis are based on standard built-in functions and are available from the lead contact upon reasonable request.
• Any additional information required to reanalyze the data reported in this study can be obtained from the lead contact upon request.
• No additional resources are reported.

## Acknowledgments

The authors gratefully acknowledge financial support by the 10.13039/100000181Air Force Office of Scientific Research, United States under award number FA9550-26-1-B114.

## Author contributions

N.Q.V. and A.K.S. designed the research program; A.K.S. performed the terahertz experiments supervised by N.Q.V.; all the authors discussed the results in the manuscript and wrote the manuscript. All authors have reviewed and approved this article. The authors declare no conflict of interest in relation to the submission of this manuscript.

## Declaration of interests

The authors declare no competing interests.

## STAR★Methods

### Key resources table

**Table:** 

REAGENT or RESOURCE	SOURCE	IDENTIFIER
**Biological samples**		

Ubiquitin from bovine erythrocytes	Sigma-Aldrich	U6253

**Software and algorithms**		

OriginPro	OriginLab	OriginPro 9.1 (64-bit)
MATLAB fitting routines	Matlab	R2026a

### Method details

#### Sample preparation

Lyophilized ubiquitin powder, with a molecular weight of ∼8.6 kDa from bovine erythrocytes, and salt-free (U6253), was procured from Sigma Aldrich, USA. Aqueous ubiquitin solutions were prepared by dissolving the protein in ultrapure water. The partial specific volumes were measured to determine the molarity of ubiquitin in the solutions.

#### Megahertz to terahertz dielectric spectroscopy

The dielectric response of 2.2 and 10.0-mM ubiquitin aqueous solutions was measured over an extended frequency range from 100 MHz to 1.12 THz using a megahertz-to-terahertz frequency-domain spectrometer. The system includes a vector network analyzer (VNA) that was interfaced with a commercial dielectric probe to measure the dynamic response of solutions in the 100 MHz – 50 GHz region. Frequency extenders from Virginia Diodes, Inc. Charlottesville, VA, were interfaced with the VNA to perform high-frequency measurements at 60 GHz – 1.12 THz.^78^

A variable path-length sample cell was employed to measure the dynamic response of aqueous samples at terahertz frequencies. High-power resistors and Peltier cooler plates (12711-5L31-03CK) from Custom Thermoelectric were installed on the sample cell body to precisely control the sample temperature. Simultaneous measurements of the absorption coefficient as well as refractive index were performed, and the corresponding dielectric response of the aqueous solutions was extracted (Figure 1).^40^^,^^43^

Temperature-dependent measurements were performed on 10.0 mM ubiquitin solutions to ensure sufficient signal-to-noise in the sub-gigahertz region for probing the hydration dynamics. Control measurements at 2.2 and 10 mM produced comparable hydration numbers and the number of water molecules in the tightly-bound layer at 25°C, indicating that neither aggregation nor misfolding is significant in this concentration range. In addition, a simple hard-sphere dispersion estimate, based on the hydrodynamic radius of ubiquitin, places the overlap concentration well above 10 mM, suggesting that hydration shells remain effectively non-overlapping at both 2.2 and 10.0 mM. Together, these observations support the use of 10.0 mM solutions for temperature-dependent analysis without introducing concentration-dependent artefacts into the extracted hydration dynamics.

#### Dielectric response of aqueous protein solutions

Dielectric measurements were performed on a frequency-domain dielectric terahertz spectrometer that covers a broad spectral range of electromagnetic waves from 100 MHz to 1.12 THz. The absorption coefficients, α(ν), and refractive indices, n(ν), were measured for pure water and aqueous protein solutions at different temperatures (Figure 1). Both quantities show a strong frequency dependence, where α(ν) monotonically increases while n(ν) decreases with rising frequency. These quantities are used to determine the complex refractive index, $n^{*}\left(\nu \right)=n\left(\nu \right)+i\alpha \left(\nu \right)c/\left(4\pi \nu \right)$, and the square root of the complex dielectric constant $\sqrt{\varepsilon_{sol}^{*}\left(\nu \right)}$ ,with c being the speed of light. The complex dielectric constant of a solution can be presented as

(Equation 5) $$\varepsilon_{\text{sol}}^{*}\left(\nu \right)=\varepsilon_{\text{sol}}^{\prime }\left(\nu \right)+i\left(\varepsilon_{\text{sol}}^{\prime \prime }\left(\nu \right)+\sigma /\left(2\pi \nu \epsilon_{0}\right)\right)\text{,}$$

where $\varepsilon_{\text{sol}}^{\prime }\left(\nu \right)$ is dielectric dispersion and $\varepsilon_{\text{sol}}^{\prime \prime }\left(\nu \right)$ is dielectric loss, respectively. The imaginary component of the dielectric response of an ubiquitin solution contains two contributions, including the dielectric loss and Ohmic loss. The Ohmic loss, $\sigma /\left(2\pi \nu \epsilon_{0}\right)$, originates from the drift of ions contained in the protein solutions, $\epsilon_{0}$ is the vacuum permittivity, and σ is the electrical conductivity of the solution. The dielectric dispersion and loss can be extracted from the refractive index and absorption coefficient through

(Equation 6) $$\begin{array}{c} \epsilon_{\text{sol}}^{\prime }\left(\nu \right)=n^{2}\left(\nu \right)-\kappa^{2}\left(\nu \right)=n^{2}\left(\nu \right)-{\left(c\alpha \left(\nu \right)/4\pi \nu \right)}^{2}\\ \epsilon_{\text{sol}}^{\prime \prime }\left(\nu \right)=2n\left(\nu \right)\cdot \kappa \left(\nu \right)=2n\left(\nu \right)c\alpha \left(\nu \right)/4\pi \nu -\sigma /\left(2\pi \nu \epsilon_{0}\right) \end{array}$$

Ubiquitin samples were obtained in lyophilized form. It should be noted that even though the protein was lyophilized and salt free, it may retain trace amounts of counterions (e.g., chloride, acetate, or phosphate) from the protein purification and/or lyophilization buffer. Common lyophilization buffers (e.g., ammonium acetate or phosphate-based buffers) may leave behind residual ions that contribute to conductivity. Furthermore, ubiquitin has charged residues (lysines, arginines, glutamates, and aspartates), and upon solvation in deionized water, some of these groups may ionize, releasing protons or hydroxyl ions. In our measurements, we observed a small but finite DC conductivity, which was accounted for by analyzing the data with an additive term in the total dielectric loss. Specifically, we followed the approach detailed in our previous works,^13^^,^^25^ where the conductivity contribution was subtracted to isolate the intrinsic dielectric relaxation of the system (Figure S1).

### Quantification and statistical analysis

The megahertz-to-terahertz dielectric response was analyzed in two parts using OriginPro (OriginLab) and custom MATLAB fitting routines. Low-frequency hydration dynamics (100 MHz - 50 GHz) were evaluated using Equation 1 in OriginPro. The real and imaginary components of the complex permittivity were fitted simultaneously using a nonlinear least-squares algorithm to minimize uncertainty in dielectric strengths and limit error propagation in hydration water estimates. All the seven parameters in Equation 1 were treated as free, except the bulk-water relaxation time *τ*_D_ which was initially fixed at 8 ps to aid convergence and then released after several iterations. The terahertz dielectric response (100 GHz - 1.12 THz) was analyzed separately to extract the dielectric response of hydrated proteins using effective-medium approximation.
