# Supplementary material for: Thermally activated coupling between protein vibrations and interfacial water dynamics revealed by terahertz spectroscopy
Source: iScience. 2026 May 25;29(6):115981. doi: 10.1016/j.isci.2026.115981 (PMC13309994; doi:10.1016/j.isci.2026.115981)

iScience, Volume *29*

Supplemental information

Thermally activated coupling between protein vibrations and interfacial water dynamics revealed by terahertz spectroscopy

Abhishek K. Singh and Nguyen Q. Vinh

Figure S1. Terahertz dielectric response of aqueous ubiquitin solution. A.) The dielectric response of 10 mM aqueous ubiquitin solution is shown with (in red) and without (in black) electrical conductivity. Also shown is the electrical conductivity of protein solutions estimated from fitting. B.) Electrical conductivity of the aqueous ubiquitin solution. In our measurements, we observed a small but finite DC conductivity, which was accounted for by analyzing the data with an additive term in the total dielectric loss. Specifically, we followed the approach detailed in our previous works, where the conductivity contribution was subtracted to isolate the intrinsic dielectric relaxation of the system.

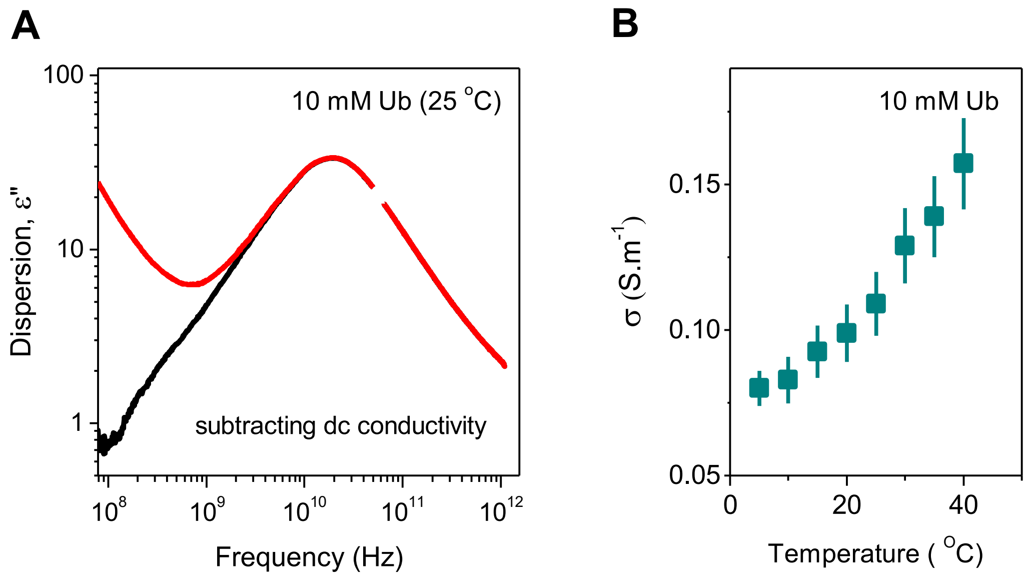

Figure S2. Hydration water dynamics at megahertz to gigahertz frequencies: Comparison of deconvolution methods. Dielectric-loss spectra of 10 mM ubiquitin solution spanning megahertz to gigahertz frequencies were fitted using different models, (A) a Debye + two Cole-Cole (D + CC_1 + CC_2) model, (B) a three-Debye model, and (C) a two-Debye model. Panels (D), (E), and (F) are residuals for each fit, respectively, underscoring their comparative accuracies. Panels (A) and (D) illustrate the dielectric response and residuals using the three-Debye model, which includes contributions from tightly-bound, loosely-bound, and bulk water, as defined in Equation 1. An alternative approach employs a Debye term for bulk water along with two Cole-Cole components to describe tightly- and loosely-bound water, $\epsilon^*_{\text{sol}}(\nu) - \epsilon_\infty = \frac{\epsilon_S - \epsilon_1}{1+(i2\pi\nu\tau_1)^{1-\alpha_1}} + \frac{\epsilon_1 - \epsilon_2}{1+(i2\pi\nu\tau_2)^{1-\alpha_2}} + \frac{\epsilon_2 - \epsilon_\infty}{1+i2\pi\nu\tau_D} + \frac{\sigma}{2\pi\nu\epsilon_0}$, where α_1 and α_2 are Cole-Cole components for spectral broadening due to distribution of relaxation times, Panels (B) and (E). It is noteworthy that setting this parameter to zero in the first and second term leads to a Debye component and the above equation reduces to a three-Debye model shown in Equation 1. To estimate the extent of distribution of relaxation times in hydration dynamics, a comparative residual analysis of dielectric loss to the two models is shown. The α_1 and α_2 are Cole-Cole parameters extracted from fitting are 0.04 and 0.06, respectively, for tightly and loosely-bound water. The convergence of α parameters close to zero shows a subtle effect of distribution of relaxation times in hydration water. In addition, no significant difference was seen in residuals from the two fittings. Also shown is a fit of the dielectric response to two-Debye model for comparison, Panels (C) and (F). This comparison supports the applicability of the three-Debye model for protein solutions, offering a simpler yet reliable fitting approach for describing hydration water dynamics.

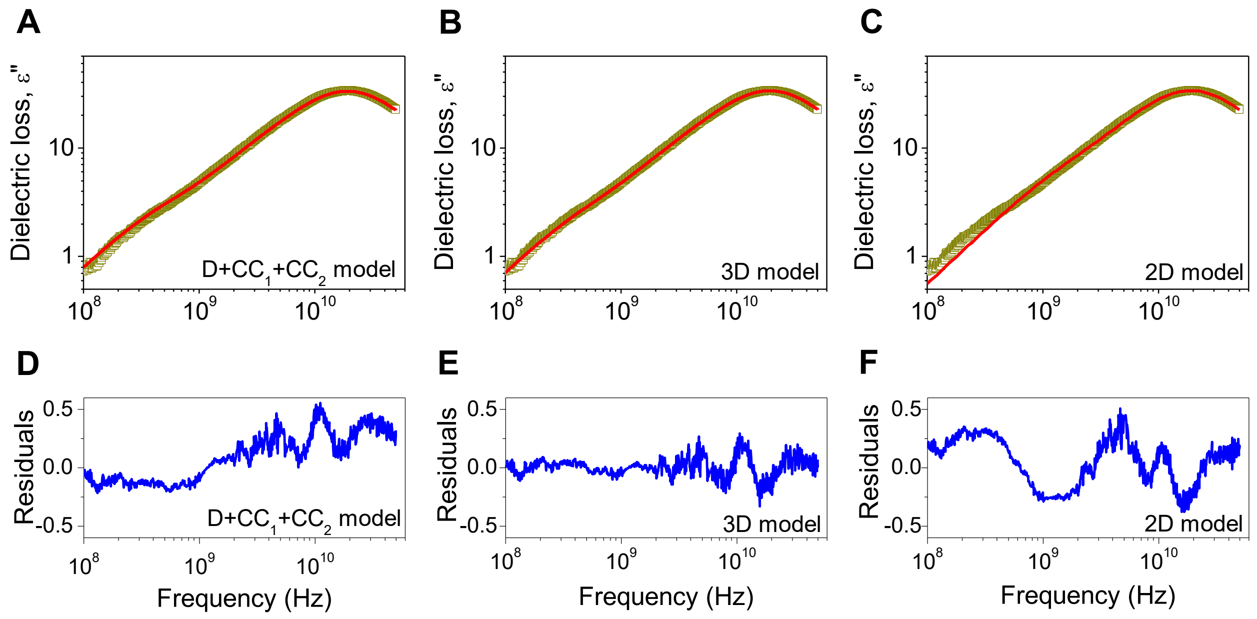

Figure S3. Effective medium approximation at terahertz frequencies. Absorbance of bare ubiquitin in 10 mM solution estimated using a two-component model. To investigate the terahertz absorption properties of protein in solution, we employed a commonly used approach from the literature, where an aqueous protein solution is treated as a two-component system consisting of "*bare*" protein and water. Under this assumption, the measured absorption coefficient of the protein solution, α_{sol}, is approximated as a concentration-weighted sum of absorption coefficients of water and bare protein and given by, $\alpha_{\mathrm{sol}} = \alpha_{\mathrm{wat}} + \alpha_{\mathrm{Ub}} = \sigma_{\mathrm{wat}} M_{\mathrm{wat}} + \sigma_{\mathrm{Ub}} M_{\mathrm{Ub}}$, where α_{wat} and α_{Ub} are the absorption coefficients of water and ubiquitin, M_{wat} and M_{Ub} are their molarities in the solution, respectively, and σ_{wat}, and σ_{Ub} are their respective molar absorption coefficients. By measuring absorption coefficients of the solution, α_{sol}, and using the known value of water, σ_{wat}, we extracted the absorbance of bare ubiquitin in a 10-mM solution. The extracted bare ubiquitin absorbance is negative between 122–361 GHz (minimum ~150 GHz), indicating that a simple two-component description is insufficient. This unphysical result reflects the presence of tightly-bound hydration water that does not contribute to terahertz absorption. Setting the minimum absorbance to zero provides a lower bound on the tightly-bound hydration shell size. Applying the Bruggeman effective-medium approximation, which assumes protein dielectric loss vanishes at zero frequency, yields a tightly-bound hydration shell of 188 ± 20 water molecules per ubiquitin, corresponding to sub-monolayer surface coverage.

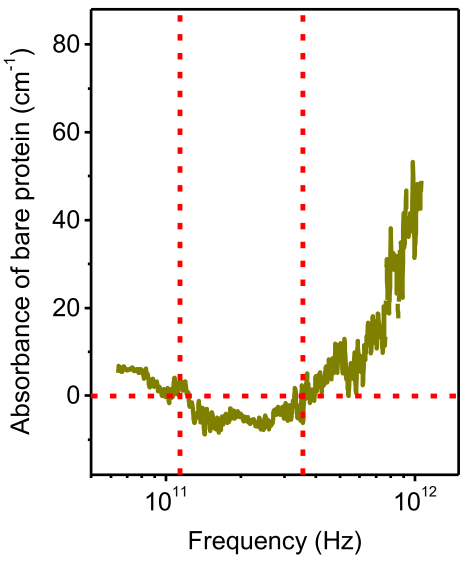

Supplement: Document S1. Figures S1–S3 [file mmc1.pdf]
